# Nursing Students’ Grit, Socio-Cognitive Mindfulness, and Achievement Emotions: Mediating Effects of Socio-Cognitive Mindfulness

**DOI:** 10.3390/ijerph19053032

**Published:** 2022-03-04

**Authors:** Mikyoung Lee

**Affiliations:** Department of Nursing, Kwangju Women’s University, Gwangju 62396, Korea; mikylee@kwu.ac.kr; Tel.: +82-62-950-3807

**Keywords:** nursing students, grit, socio-cognitive mindfulness, achievement emotions

## Abstract

Background: Recognizing the under-examined socio-cognitive mindfulness and achievement emotions in nursing, this study aimed to examine the relationships between grit, socio-cognitive mindfulness, and achievement emotions among nursing students, as well as the mediating effects of socio-cognitive mindfulness. Methods: This study utilized a cross-sectional design. A total of 220 nursing students in Korea completed the questionnaire measuring the levels of grit, socio-cognitive mindfulness, and achievement emotions. To analyze data, structural equation modeling and path analysis were performed. Results: Grit was positively related to socio-cognitive mindfulness and positive achievement emotions but negatively related to negative emotions. Socio-cognitive mindfulness was positively related to positive emotions but negatively related to negative emotions. In addition, the mediating effects of socio-cognitive mindfulness were found in the association between grit and achievement emotions in nursing students. Conclusions: Grittier students tend to have higher socio-cognitive mindfulness and positive emotions but lower negative emotions in learning environments. Mediating effects highlight the benefits of socio-cognitive mindfulness in the context of nursing education, providing the basis for developing practical mindfulness programs to cultivate nursing students’ socio-cognitive mindfulness.

## 1. Introduction

Nursing students might be seen as having a stable college life because of having a relatively clear goal as to their future career and a higher employment rate compared to students in other majors; however, in actuality, they constantly report that they are experiencing more stress and negative emotions due to a heavy study load, frequent exams, and clinical practice compared to other major students [1,2]. It is critical to pay attention to nursing students’ psychological well-being, focusing on emotional experiences, which directly influence their learning and college life [2]. Thus, it is important to identify the factors influencing nursing students’ emotions and accordingly prepare strategies for improving their emotional experiences.

Achievement emotions are learners’ specific emotions that are directly tied to achievement activities (e.g., studying) or achievement outcomes (success and failure); they are omnipresent in educational settings [3]. For example, students often experience a variety of emotions such as enjoyment, hope, pride, anxiety, anger, frustration, boredom, shame, or hopelessness in learning environments. Acknowledging that achievement emotions can have profound influences on students’ learning, academic performance, and psychological well-being, researchers have conducted considerable studies on them in the education field [4,5,6]. However, research on achievement emotions is still in the early stages of research in the nursing field, although consideration of nursing students’ emotions should be of more concern in comparison to those of students in other majors [2]. Not only to deal with considerable stress and negative emotions but also to improve empathy skills, which is one of the key competencies of nurses, nursing students should first be able to recognize their own emotions. Limited literature has found that nursing students’ positive achievement emotions are positively related and negative achievement emotions are negatively related to mindfulness and academic outcomes [2,7]. In addition, nursing students’ positive achievement emotions are positively related to a reappraisal strategy (cognitive change to modify emotions) of emotion regulation, and negative achievement emotions are positively related to a suppression strategy (response modification) [8].

Grit and mindfulness could be influential elements among various factors that affect students’ emotions. In particular, grit has been recognized as one of the important psychological constructs that influence students’ learning and academic achievement [9,10,11]. Grit is defined as “perseverance and passion for long-term goals” [12] (p. 1087). It consists of two factors, consistency of interest and persistency of effort, for achieving those long-term goals [12]. Individuals with a high level of grit work hard toward challenges, and they keep investing effort and interest over time despite failures and difficult situations in progress; consequently, they are likely to exhibit greater psychological well-being [9,12]. In particular, for nursing students who require constant effort and passion to successfully manage their extensive study load and clinical practice, grit could be an essential variable for academic adaptation and outcomes [13].

Mindfulness has been known as one of the factors related to grit [14,15,16] and emotions [2,14,17]. There are two main frameworks for mindfulness: meditative mindfulness with Kabat-Zinn’s [18] Eastern perspective and socio-cognitive mindfulness with Langer’s [19] Western social psychological perspective. Meditative mindfulness is defined as “awareness that emerges through paying attention on purpose, in the present moment, and nonjudgmentally to the unfolding of experience moment by moment” [20] (p. 145). In comparison, socio-cognitive mindfulness is defined as “a flexible state of mind in which one is anchored in the present moment by engaging with novel ideas, while paying attention to context and variability” [21] (p. 62). Socio-cognitive mindfulness emphasizes openness to novelty that results in awareness to distinction and sensitivity to different contexts with multiple perspectives [21,22,23]. This can promote students’ engagement in a learning agenda, problem-solving, creativity, and other cognitive exercises by increasing their attention and awareness [22]. This socio-cognitive mindfulness approach by Langer was adopted in this study.

Researchers in several disciplines, including nursing, have placed enormous interest in meditative mindfulness for years, revealing that meditative practices were effective in reducing stress [24] and unpleasant emotions [25] or improving emotions and emotional regulation [26]. However, socio-cognitive mindfulness has just started gaining nursing researchers’ interest. Pioneering studies on nursing students’ socio-cognitive mindfulness emphasized the positive effects of socio-cognitive mindfulness in nursing education. For example, nursing students with higher socio-cognitive mindfulness rated their communication self-efficacy higher than students with lower mindfulness [27]. Regarding the association between socio-cognitive mindfulness and emotions, nursing students’ socio-cognitive mindfulness was related to improving positive achievement emotions and reducing negative emotions [2,8]. In addition, nursing students’ socio-cognitive mindfulness was positively related to a reappraisal strategy and negatively related to a suppression strategy in terms of emotion regulation [8].

As the psychological constructs of grit and mindfulness have gained researchers’ attention in recent years [15], a few studies investigating the relationship between grit and meditative mindfulness were published [14,15,16]. These studies reported a positive correlation between grit and meditative mindfulness. However, to the best of my knowledge, there is no research on how grit and socio-cognitive mindfulness are associated with each other in the nursing field. Meanwhile, previous research has found that grit was positively associated with students’ positive emotions but negatively associated with negative emotions [14,16,28,29]. Nevertheless, the mechanisms of the influence of grit on students’ achievement emotions have not been explored in the literature. In particular, there has been no empirical research to directly test the mediating function of socio-cognitive mindfulness in the link between grit and achievement emotions in nursing. Finally, concerning research on socio-cognitive mindfulness and emotions, socio-cognitive mindfulness was positively related to positive emotions [30] and negatively related to negative emotions [30,31,32]. Recent studies with nursing students identified a positive correlation between socio-cognitive mindfulness and positive achievement emotions and a negative correlation between mindfulness and negative achievement emotions [2,8].

**Figure 1:**
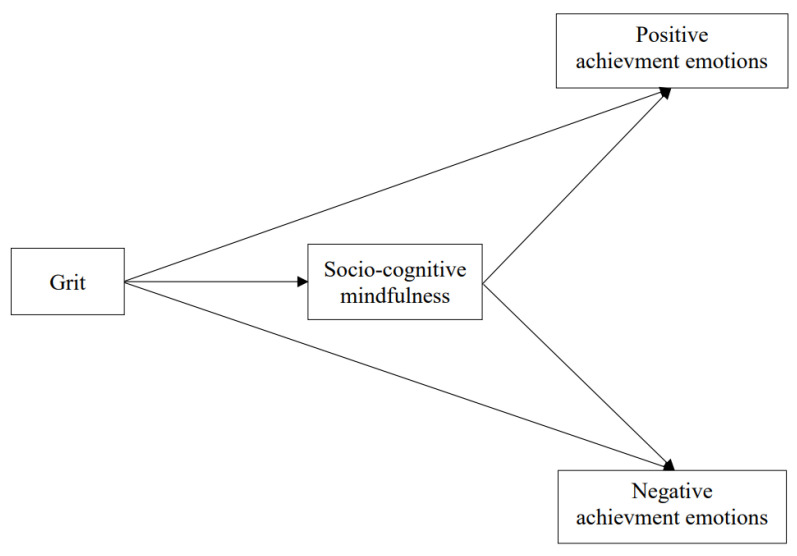
Conceptual framework.

The present study paid special attention to the under-examined socio-cognitive mindfulness and achievement emotions in nursing. This study aimed to examine the relationships between grit, socio-cognitive mindfulness, and achievement emotions among nursing students. Furthermore, this study investigated the mediating effects of socio-cognitive mindfulness in the relationship between grit and achievement emotions. Founded on the previous literature above, the following hypotheses have been proposed, and the conceptual framework of the present study is illustrated in Figure 1.

**Hypothesis 1 (H1).**  *Grit is positively related to socio-cognitive mindfulness.*

**Hypothesis 2 (H2).**  *Grit is positively related to positive achievement emotions but negatively related to negative achievement emotions.*

**Hypothesis 3 (H3).**  *Socio-cognitive mindfulness is positively related to positive achievement emotions but negatively related to negative achievement emotions.*

**Hypothesis 4 (H4).**  *Socio-cognitive mindfulness mediates the relationship between grit and achievement emotions.*

## 2. Materials and Methods

### 2.1. Research Design

This study utilized a cross-sectional design to investigate the relationships between grit, socio-cognitive mindfulness, and achievement emotions as well as the mediating effects of socio-cognitive mindfulness.

### 2.2. Participants and Procedure

The participants in this study were 220 female nursing students at a women’s university in a metropolitan city in South Korea. They consisted of 102 sophomores (46.4%) and 118 juniors (53.6%), with a mean age of 21.03 (SD = 2.76). The sample size of the present study was considered based on the Chou and Bentler’s [33] recommendation. They argue that a sample size of 200 or more is desirable when utilizing a maximum likelihood in structural equation modeling (SEM). Thus, the present sample size of 220 was suitable for the research model in this study. Freshmen were not included because the achievement emotions variable was examined in terms of nursing major subjects, and nursing students start taking major-specific subjects in the second year. Senior students were also excluded to protect the vulnerable group because the author was involved in teaching them.

Data were collected between 2 and 31 August 2021 online due to the COVID-19 pandemic situation in Korea. The participants read the description about the research and understood the purpose and importance of the research. They were reassured that their information would be kept confidential and used only for the purpose of research. They were also informed that they could discontinue participating in the study at any time if they so wished. Then, they voluntarily consented to participate in the study and started answering the questionnaire. The questionnaire consisted of the scales of grit, socio-cognitive mindfulness, and achievement emotions, and demographic information. A research assistant provided a small gift of stationery to the participants in September when in-person instruction resumed at the university.

### 2.3. Ethical Considerations

This study was approved by the Institutional Review Board at the participants’ university in Korea (1041465-202105-HR-001-20). Approval from the dean of the nursing department and the professors of the participants’ classes was also received. Students answered the questionnaire on a volunteer basis. They were also allowed to stop at any time without any penalty if they felt uncomfortable while responding to the questionnaire.

### 2.4. Measures

#### 2.4.1. Grit

The Grit Scale developed by Duckworth et al. [12] was used to measure nursing students’ grit. For the present participants, the Korean validated version of the Grit Scale by Lee [34] was used. This scale evaluates two elements of grit with 12 items: six items each relating to consistency of interests (e.g., “I often set a goal but later choose to pursue a different one”) and perseverance of effort (e.g., “I have overcome setbacks to conquer an important challenge”). Items for consistency of interests were reverse coded. Participants answered on a five-point Likert scale of 1 (strongly disagree) to 5 (strongly agree). The total score of the Grit Scale was used in the present analyses, given that the total score actually demonstrated a more comprehensive assessment of the grit construct [12,35]. Cronbach’s alpha was 73 for the total scale; for consistency of interests and perseverance of effort, Cronbach’s alphas were 72 and 68, respectively, presenting adequate internal reliability.

#### 2.4.2. Socio-Cognitive Mindfulness

The Langer Mindfulness Scale (LMS) developed by Bodner and Langer [36] was adopted to assess socio-cognitive mindfulness. For the present sample, the Korean validated version of LMS by Kim [37] was used. The LMS consists of 21 items to assess four sub-categories of socio-cognitive mindfulness. The four sub-categories are novelty seeking (six items; e.g., “I like to investigate things”), novelty producing (six items; e.g., “I try to think of new ways of doing things”), flexibility (four items; e.g., “I am always open to new ways of doing things”), and engagement (five items; e.g., “I get involved in almost everything I do”). All the items were answered on a five-point Likert scale of 1 (strongly disagree) to 5 (strongly agree). The total score of the LMS was used because the aim of the study was to examine the relationships between the main constructs of grit, socio-cognitive mindfulness, and achievement emotions. In addition, some researchers have suggested that using the total score of the mindfulness scale was effective in keeping the analytic procedure as parsimonious as possible [38,39]. Cronbach’s alpha was 88 for the total scale. Regarding the sub-categories, Cronbach’s alphas were 73, 80, 60, and 69 for novelty seeking, novelty producing, flexibility, and engagement, respectively, suggesting acceptable internal reliability.

#### 2.4.3. Achievement Emotions

To measure the participants’ achievement emotions regarding the nursing studies, the Korean Achievement Emotions Questionnaire (AEQ) for Korean nursing students [7] was utilized. The original AEQ was developed by Pekrun et al. [40], and the Korean AEQ used in this study was a modified version for nursing students of the Korean validated AEQ [41]. This measure evaluates three positive emotions (enjoyment, hope, and pride) and five negative emotions (boredom, anger, anxiety, hopelessness, and shame), consisting of 12 positive emotion items (e.g., “I am looking forward to my classes”) and 20 negative emotion items (e.g., I worry whether I will be able to understand all the materials). Participants responded on a five-point Likert scale of 1 (strongly disagree) to 5 (strongly agree). Cronbach’s alphas for positive emotions and negative emotions were 86 and 90, respectively, exhibiting satisfactory internal reliability.

### 2.5. Data Analyses

Correlations, means, and standard deviations for the study variables were analyzed with a SPSS 28 program [IBM Corporation, Armonk, NY, USA]. To test the hypotheses of the relationships between grit, socio-cognitive mindfulness, and achievement emotions, structural equation modeling (SEM) using the Mplus 8 program [42] was performed. The model fit was evaluated with the comparative fit index (CFI), the root-mean-square-error of approximation (RMSEA), the Tucker–Lewis Index (TLI), and the standardized root-mean-square-residual (SRMR) by the rules of CFI > 0.90, TLI > 0.90 [43] and RMSEA < 0.080 and SRMR < 0.080 [44]. Furthermore, path analysis with Mplus 7 was conducted to explore the mediating effects of socio-cognitive mindfulness in the relationship between grit and achievement emotions.

## 3. Results

### 3.1. Preliminary Results

Table 1 presents correlations, means, and standard deviations for the study variables. The mean of the nursing students’ grit level was 2.95 (SD = 0.48) out of 5. The mean level of socio-cognitive mindfulness was 3.42 (SD = 0.46). Regarding achievement emotions, participants reported higher positive emotions (M = 3.30, SD = 0.54) than negative emotions (M = 2.75, SD = 0.61). There were positive correlations between all of the study variables except for negative achievement emotions: between grit and socio-cognitive mindfulness (r = 0.310, *p* < 0.01), between grit and positive achievement emotions (r = 0.433, *p* < 0.01), and between socio-cognitive mindfulness and positive achievement emotions (r = 0.607, *p* < 0.01). On the other hand, the negative achievement emotions were negatively related to grit (r = −0.465, *p* < 0.01), socio-cognitive mindfulness (r = −0.370, *p* < 0.01), and positive achievement emotions (r = −0.625, *p* < 0.01).

### 3.2. Relationships between Grit, Socio-Cognitive Mindfulness, and Achievement Emotions (Hypotheses 1−3)

**Figure 2:**
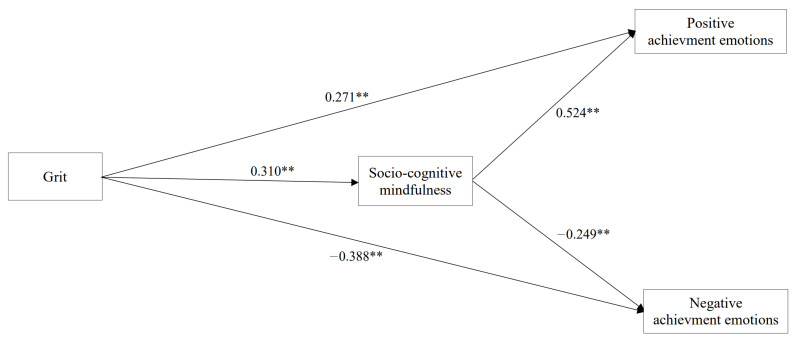
Structural equation model displaying parameter estimates for effects of grit on socio-cognitive mindfulness and achievement emotions. ** *p* < 0.01.

SEM using Mplus 8 was conducted to investigate the relationships between grit, socio-cognitive mindfulness, and achievement emotions among the nursing students. The model was saturated, demonstrating CFI = 1.000, TLI = 1.000, RMSEA = 0.000, and SRMR = 0.000. This suggests that the present research model fits the data perfectly. Figure 2 displays path coefficients among grit, socio-cognitive mindfulness, and achievement emotions.

First, grit positively influenced socio-cognitive mindfulness (β = 0.310, *p* < 0.01) (Hypothesis 1). Second, regarding the associations between grit and achievement emotions (Hypothesis 2), grit was positively related to positive emotions (β = 0.271, *p* < 0.01) but negatively related to negative emotions (β = −0.388, *p* < 0.01). Third, socio-cognitive mindfulness was positively related to positive emotions (β = 0.524, *p* < 0.01) but negatively related to negative emotions (β = −0.249, *p* < 0.01) (Hypothesis 3).

### 3.3. Mediating Effects of Socio-Cognitive Mindfulness (Hypothesis 4)

Path analysis using Mplus 8 was performed to investigate whether socio-cognitive mindfulness would mediate the association between grit and achievement emotions. Mediation analyses revealed that socio-cognitive mindfulness played a mediating role in the relationship between the nursing students’ grit and achievement emotions. The details of the total, direct, and indirect effect (i.e., mediating effect) are shown in Table 2.

In the association between grit, socio-cognitive mindfulness, and positive achievement emotions, grit was positively associated with socio-cognitive mindfulness (a = 0.310, *p* < 0.01), and socio-cognitive mindfulness was positively associated with positive emotions (b = 0.524, *p* < 0.01). The direct effect of grit on positive achievement emotions was reduced after controlling for the socio-cognitive mindfulness effect (c′ = 0.271, *p* < 0.01), in comparison with the total effect (c = 0.433, *p* < 0.01). The indirect effect (mediating effect) through socio-cognitive mindfulness was significant (a × b = 0.162, *p* < 0.01). This indicates that socio-cognitive mindfulness partially mediates the relationship between grit and positive emotions.

In the link between grit, socio-cognitive mindfulness, and negative achievement emotions, grit was positively related to socio-cognitive mindfulness (a = 0.310, *p* < 0.01), and socio-cognitive mindfulness was negatively related to negative emotions (b = −0.249, *p* < 0.01). The direct effect of grit on negative achievement emotions decreased after controlling for the socio-cognitive mindfulness effect (c′ = −0.388, *p* < 0.01), in comparison with the total effect (c = −0.465, *p* < 0.01). The indirect effect through socio-cognitive mindfulness was significant (a × b = −0.077, *p* < 0.01). This suggests that socio-cognitive mindfulness partially mediates the relationship between grit and negative emotions.

## 4. Discussion

The purpose of the study was to examine the relationships between grit, socio-cognitive mindfulness, and achievement emotions among nursing students in Korea, as well as the mediating effects of socio-cognitive mindfulness in the relationship between grit and achievement emotions. The findings showed that grit was positively related to socio-cognitive mindfulness and positive achievement emotions but negatively related to negative emotions. Socio-cognitive mindfulness was positively related to positive emotions but negatively related to negative emotions. Finally, the mediating effects of socio-cognitive mindfulness were found in the association between grit and achievement emotions.

First, the participants with a higher level of grit reported a higher level of socio-cognitive mindfulness, supporting Hypothesis 1. Although research focusing on socio-cognitive mindfulness is limited regarding the relationship of grit with mindfulness, several studies have found that grit and meditative mindfulness are positively related [14,15,16]. Given that both meditative and socio-cognitive mindfulness perspectives share similar characteristics despite having some different features, the present result of a positive correlation between grit and socio-cognitive mindfulness could be explained. For example, researchers have agreed that both mindfulness frameworks accentuate awareness, focused attention, and openness to new experiences with flexibility and curiosity, and that both perspectives acknowledge acceptance of the present moment through self-regulation and de-automatization [31,45,46]. In fact, earlier literature demonstrated a strong positive correlation between both meditative and socio-cognitive mindfulness [2,46,47].

The current finding indicates that the grittier students might have become more mindful in the nursing education context. That is, when nursing students are constantly interested in their assigned tasks, and they continue to make an effort even under challenging situations, they are more likely to concentrate on the present moment with increased attention and awareness to accomplish their goals; this describes an important element of socio-cognitive mindfulness [21,22,23]. As Yeh et al. [48] claimed that socio-cognitive mindfulness-based learning should include the grit construct and emotion regulation, this study expands research on mindfulness and grit by generating a pioneering result of a significant association between grit and socio-cognitive mindfulness among nursing students.

Second, regarding the associations between grit and achievement emotions, grit was positively related to positive emotions but negatively related to negative emotions, confirming Hypothesis 2. Consistent with past work, the current finding supports the idea that grittier individuals tend to have a higher level of positive emotions and a lower level of negative emotions [14,16,28,29,49]. The current finding extends on this previous research by focusing on nursing students’ specific achievement emotions in the learning context. This finding also supports the evidence that grittier people have a more positive personality profile of lower neuroticism and higher extraversion [9], which are known to be associated with emotional well-being [50]. Considering the two facets of grit (i.e., consistency of interest and persistency of effort), when students are constantly interested and thus become engaged in what they are working on, they are likely to experience more positive emotions and less negative emotions. Furthermore, when students persist more to complete their tasks, they are likely to accomplish more and thereby feel the reward of greater accomplishment and have more pleasant emotions [16]. These previous findings are also reflected in the present result.

Third, nursing students’ socio-cognitive mindfulness was positively associated with positive achievement emotions but negatively associated with negative emotions related to nursing studies, supporting Hypothesis 3. This is in line with the previous findings that socio-cognitive mindfulness helped students experience more positive emotions by allowing them to become engrossed in their tasks through cognitive flexibility [51,52]. The result is also supported by previous findings of a positive correlation between socio-cognitive mindfulness and positive emotions [30] and a negative correlation between socio-cognitive mindfulness and negative emotions [30,31,32]. Additionally, through socio-cognitive mindfulness, the nursing students were able to enhance their emotional experiences by leading to greater communication self-efficacy and empathy skills [27]. In particular, the present result is repetitive of recent studies conducted with a sample of nursing students in Korea [2,8]. The current finding reinforces the idea that individuals equipped with a higher level of socio-cognitive mindfulness pay more attention to the present moment and thereby improve cognitive flexibility and insights [53], which facilitates students’ flexible thinking, learning skills, and cognitive outcomes. This would ultimately promote students’ positive emotions and reduces negative emotions [2,8,51].

Finally, the mediating results support Hypothesis 4, that is, that socio-cognitive mindfulness mediates the positive association between grit and positive achievement emotions as well as the negative association between grit and negative achievement emotions in nursing students. This mediation indicates both the direct influence of grit and the indirect influence of socio-cognitive mindfulness on achievement emotions. This means that grittier students may possess a higher level of socio-cognitive mindfulness, thus experiencing more positive emotions but less negative emotions. Previous literature has reported mediating roles of meditative mindfulness in the association between nurses’ self-esteem and burnout [54], between medical interns’ coping strategies and stress [55], between students’ grit and life satisfaction [14], and between students’ anxiety and psychological well-being [56]. However, the current study is the initial research on the mediating role of socio-cognitive mindfulness between grit and achievement emotions among nursing students.

Based on the mediating result, it is assumed that socio-cognitive mindfulness might be one mechanism to account for the link between nursing students’ grit and achievement emotions. The mediation effects were found to be partial, suggesting that mindfulness accounts for only a part of the relationship between grit and achievement emotions. Thus, there still exists other unexplained pathways linking grit to achievement emotions. Future research might explore the mechanism that links grit to achievement emotions in more detail. In short, this highlights the importance of socio-cognitive mindfulness in the context of nursing education, encouraging empirical research on students’ socio-cognitive mindfulness in nursing. Notably, this is the first empirical evidence revealing that a high level of socio-cognitive mindfulness helps to explain the reason for the significant connection between nursing students’ grit and their achievement emotions. It will be worthwhile to expand this research with a longitudinal design to more thoroughly inspect socio-cognitive mindfulness as a mediator.

Some limitations of this study are as follows. First, the current study used only cross-sectional data; thus, the causal relationships between the variables should be cautiously interpreted. Future research should include a longitudinal or experimental design to more comprehensively assess the causal relationships between grit, socio-cognitive mindfulness, and achievement emotions. Second, only self-reported data was utilized in this study, which might have produced biased results. In particular, when students were asked about their achievement emotions, they might have expressed desirable emotions rather than the emotions they actually felt. Future studies may consider qualitative research such as conducting interviews or class observations or collecting data from peers to attain more objective data. Third, the present sample included only female nursing students in one university in Korea; thus, it might be difficult to generalize the results to other majors, gender, or countries. To replicate the present findings and expand this research, further studies should involve diverse samples in terms of majors, gender, and nationalities.

## 5. Conclusions

Acknowledging under-examined constructs of socio-cognitive mindfulness and achievement emotions in nursing education, the present research confirmed significant relationships between grit, socio-cognitive mindfulness, and achievement emotions among nursing students. In particular, this is the first investigation on the mediating role of socio-cognitive mindfulness in the association between nursing students’ grit and achievement emotions, revealing the benefits of socio-cognitive mindfulness in the context of nursing education. The present findings can provide the basis for developing practical mindfulness programs to cultivate nursing students’ socio-cognitive mindfulness. The socio-cognitive mindfulness program can ultimately help nursing students improve positive emotions and diminish negative emotions in learning environments, which will potentially enhance their psychological well-being and their college experience.

## Figures and Tables

**Table 1:** Correlations, means, and standard deviations for the study variables (N = 220).

Variables	1	2	3	4
1. Grit	1			
2. Socio-cognitive mindfulness	0.310 **	1		
3. Positive emotions	0.433 **	0.607 **	1	
4. Negative emotions	−0.465 **	−0.370 **	−0.625 **	1
Mean *^a^*	2.95	3.42	3.30	2.75
*SD*	0.48	0.46	0.54	0.61

Note. *^a^* Possible range 1–5. ** *p* < 0.01.

**Table 2:** Socio-cognitive mindfulness as mediators of effects of grit on achievement emotions (N = 220).

IV	M	DV			Total Effect	Direct Effect	Indirect Effect
			IV→M (a)	M→DV (b)	IV→DV (c)	IV→DV (c′)	IV→M→DV
							(a × b)
Grit	SCMF	Positive emotions	0.310 **	0.524 **	0.433 **	0.271 **	0.162 **
Grit	SCMF	Negative emotions	0.310 **	−0.249 **	−0.465 **	−0.388 **	−0.077 **

Note. IV = Independent variable; M = Mediator; DV = Dependent variable; SCMF = Socio-cognitive mindfulness. Standardized coefficients are reported. ** *p* < 0.01.

## Data Availability

The data presented in this study are available upon request from the corresponding author.
